# *Megasphaera elsdenii*, a commensal member of the gut microbiota, is associated with elevated gas production during *in vitro* fermentation

**DOI:** 10.1017/gmb.2023.18

**Published:** 2023-12-21

**Authors:** Erasme Mutuyemungu, Hollman A. Motta-Romero, Qinnan Yang, Sujun Liu, Sean Liu, Mukti Singh, Devin J. Rose

**Affiliations:** 1 Department of Food Science and Technology, University of Nebraska–Lincoln, Lincoln, NE, USA; 2 Nebraska Food for Health Center, University of Nebraska–Lincoln, Lincoln, NE, USA; 3 Research & Development, Isolation Bio, San Carlos, CA, USA; 4 Project Management, Chobani, Twin Falls, ID, USA; 5 Department of Microbiology & Immunology, University of Michigan, Ann Arbor, MI, USA; 6 Functional Food Research Unit, USDA, Agricultural Research Service, National Center for Agricultural Utilization Research, Peoria, IL, USA; 7 Department of Agronomy and Horticulture, University of Nebraska–Lincoln, Lincoln, NE, USA

**Keywords:** flatulence, bloating, short chain fatty acids, kidney beans, sweet potatoes

## Abstract

*Megasphaera elsdenii* has been correlated with gas production by human faecal microbiota during fermentation. The objective of this study was to determine the role of *M. elsdenii* in gas production by the microbiome. Kidney beans and sweet potatoes were subjected to *in vitro* digestion and dialysis followed by fermentation with ten faecal microbiomes: three with detectable *M. elsdenii* (Me_D) and seven with no detectable *M. elsdenii* (Me_ND). Me_D microbiomes produced more gas than the Me_ND microbiomes (*p* < 0.001). Me_D microbiomes produced more gas during fermentation of sweet potatoes than kidney beans (*p* < 0.001), while the opposite was true for the Me_ND microbiomes (*p* < 0.001). Among amplicon sequence variants that were associated with gas production, *M. elsdenii* had the strongest association (*p* < 0.001). Me_D microbiomes consumed more acetate and produced more butyrate than Me_ND microbiomes (*p* < 0.001). Gas production by *M. elsdenii* was confirmed by fermentation of sweet potatoes and acetate with human and rumen *M. elsdenii* isolates. The human isolate produced gas on sweet potatoes and acetate. This study suggests that *M. elsdenii* may be involved in gas production during the fermentation of flatulogenic foods through utilisation of undigestible substrates or cross-feeding on acetate.

## Introduction

Often flatulence is a regular, everyday occurrence that is only awkward in social circumstances where it is perceived as embarrassing. However, this can be a sufficient deterrent to the consumption of some foods that are perceived to cause flatulence (Szczebyło et al., 2020). Furthermore, sometimes intestinal gas becomes severe and can cause distress, discomfort, and chronic conditions such as irritable bowel syndrome (MacDermott, 2007). Indeed, the discomfort caused by intestinal gas production is the primary reason cited for people to visit a gastroenterologist (Manichanh et al., 2014).

The volume of intestinal gas produced by the gut microbiome varies among adult men and women, ranging from as little as 0.2 L/d to as much as 1.5 L/d (Serra et al., 1998; Suarez et al., 1998; Mego et al., 2015). The main factors influencing the variation in intestinal gas volume produced among individuals are diet and gut microbiota composition (Manichanh et al., 2014). Many people associate the intake of certain foods with high gas production (Mego et al., 2015). Typically these foods are high in dietary fibres (Bolin and Stanton, 2003). Dietary fibres escape digestion and absorption in the small intestine and are fermented by gut bacteria in the colon to yield the flatus gases: methane, hydrogen, and carbon dioxide (Mego et al., 2015).

In this study, we compared gas production between two foods that are historically associated with gas production but contain very different composition, pinto beans and sweet potatoes (Den et al., 1986; Price and Fenwick, 1988). In particular, pinto beans are high in raffinose family oligosaccharides (25 g/kg [Wang et al., 2010]), a class of non-digestible carbohydrates often implicated in the production of intestinal gas (Elango et al., 2022), while sweet potatoes lack these components. Total dietary fibre and soluble and insoluble fibres also tend to be higher in pinto beans (total: 222 g/kg dry basis; soluble: 55.3 g/kg; insoluble: 167 g/kg; [Wang et al., 2010]) compared with sweet potatoes (total: 92.7 g/kg dry basis; soluble: 28.1 g/kg; insoluble: 64.6 g/kg [Huang et al., 1999]). In contrast, sweet potatoes contain much more total and resistant starch (total: 645 g/kg dry basis; resistant: 240 g/kg [Bodjrenou et al., 2023]) than pinto beans (total: 425 g/kg; resistant: 39.8 g/kg [Wang et al., 2010]).

Many bacteria in the human gut are responsible for flatulogenic gases produced during the fermentation of non-digestible substrates in flatulence-causing foods. These bacteria mostly belong to the *Bacteroidota* and *Bacillota* phyla (formerly *Bacteroidetes* and *Firmicutes*, respectively), which together usually make up 90% or more of the total bacterial population in the human gut (Arumugam et al., 2011; Rinninella et al., 2019). However, there may be “keystone” members of the microbiota that, if present, are responsible for unusually high gas production relative to microbiomes lacking these keystone members (Banerjee et al., 2018).

In support of this speculation, in a previous study we quantified gas production during fermentation of processed pulses using faecal microbiotas from healthy donors (Rose et al., 2021). There were three amplicon sequence variants (ASV) belonging to *Clostridium sensu stricto* cluster 1, *Dialister*, and *Megasphaera elsdenii* that were significantly correlated with gas production during fermentation. The *M. elsdenii* ASV, which we termed *M. elsdenii* 4415, for the first four characters of the feature ID generated from QIIME 2 (Bolyen et al., 2019), was particularly interesting. One microbiome that contained this ASV at 20% abundance in the faecal sample and produced about 2-fold more gas compared with the other microbiomes. Additionally, the abundance of *M. elsdenii* 4415 in this microbiome increased from 20% to 50–75%, depending on substrate, during the fermentation. The abundance of the other ASVs belonging to *Clostridium sensu stricto* cluster 1 and *Dialister* were more common among all microbiomes and present at much lower abundances throughout fermentation. Thus, we speculated that *M. elsdenii* 4415 may be a keystone member of the microbiota responsible for elevated gas production during fermentation.

*M. elsdenii*, a member of the *Bacillota* phylum, is a gram-negative coccoid-shaped obligate anaerobe and has been used as a probiotic in ruminants because of its ability to metabolise lactate to short chain fatty acids (SCFA) in the prevention of rumen acidosis (Aikman et al., 2011; Shetty et al., 2013; Chen et al., 2019). However, some studies examining the efficacy of *M. elsdenii* as a probiotic feed supplement have also measured gas production and found that it is increased in animals supplemented with *M. elsdenii* (Sedighi and Alipour, 2019). *In vitro* fermentation using horse gut microbiome has shown that addition of *M. elsdenii* causes a significant increase in gas production during fermentation of inulin and corn starch (Douthit et al., 2019).

*M. elsdenii* is only sparsely present among human gut microbiotas (Duncan et al., 2004). While *M. elsdenii* may offer health benefits to hosts that harbour it due to its fermentation of dietary substrates to SCFA, including butyrate, a beneficial microbial metabolite (Shetty et al., 2013), it may also cause increased intestinal gas production and lead to intestinal discomfort (Rose et al., 2021). Therefore, the purpose of this study was to investigate the role of *M. elsdenii* in gas production during *in vitro* fermentation of two flatulogenic foods: red kidney beans and sweet potatoes.

## Materials and methods

### Faecal sample selection

Our previous study identified one ASV that was particularly interesting for its relationship to gas production (Rose et al., 2021). The Quantitative Insights into Microbial Ecology (QIIME) ID assigned to this ASV was 44158349d8858abc6c04aada0c131da5 and it was classified as *M. elsdenii*; therefore, it was named “*M. elsdenii* 4415,” assigning the first four characters of its QIIME ID.

For faecal sample selection, we examined sequencing data from 30 faecal samples for the presence of *M. elsdenii* 4415 (Supplementary Table S1). The 30 faecal samples were collected from donors with no history of gastrointestinal disorders and no probiotic supplement or antibiotic use within the last six months. The donors’ ages ranged from 21 to 60 years old (30.2 ± 9.4 years) and there were 16 males and 14 females. There were three microbiomes that had detectable *M. elsdenii* 4415 (Me_D) in faecal samples. These faecal samples were from two males, aged 25 and 27 years, and one female, aged 24 years). There were no other ASVs that classified as *M. elsdenii* among all 30 microbiomes. Seven microbiomes with no detectable *M. elsdenii* 4415 (Me_ND) were selected from among the remaining 27 to serve as a control group. These selected microbiomes represented three males and four females with ages ranging from 21 to 28 years (similar to Me_D microbiomes, *p* = 0.9). Because the purpose of this study was to examine the effects of *M. elsdenii* 4415 on gas production during the fermentation of sweet potatoes and kidney beans, the seven microbiomes that made up the Me_ND group were selected based on the diverse diets of the faecal donors with similar intakes of potatoes and legumes compared to the Me_D faecal donors. Dietary intakes of the faecal donors were estimated from responses to the Diet History Questionnaire III (National Cancer Institute – Division of Cancer Control and Population Sciences, 2022), which all stool donors completed at the time of faecal collection.

The faecal samples were prepared by mixing each faecal sample (10 g) separately with sterile anaerobic phosphate-buffered saline, pH 7.0, containing 10% glycerol as a cryoprotectant at 1:9 w/v inside a sterile filter bag (Filtra-Bag, Thomas Scientific, Swedesboro, NJ) within 2 h of defecation. A stomacher was used to homogenise each faecal slurry for 4 min, and then the mixture was transferred to an anaerobic chamber (containing 5% H_2_, 5% CO_2_, and 90% N_2_ and 0.5 m^3^ of working space, Bactron X, Sheldon Manufacturing, Cornelius, OR) and aliquoted in 15 mL polypropylene centrifuge tubes. Faecal slurries were then stored at −80°C until further use. The procedures involving human subjects were approved by the Institutional Review Board of the University of Nebraska before initiating the study (20210621206EP, 20200219980FB). All faecal donors provided written informed consent before initiating the study protocols.

### Substrate preparation

White flesh sweet potatoes (*Ipomoea batatas*) and red kidney beans (*Phaseolus vulgaris*) were purchased from a local market. Sweet potatoes were cooked as previously described with some modifications (Bengtsson et al., 2009). Specifically, the sweet potatoes were peeled, cut into cubic pieces (2.0 cm × 2.5 cm), and covered with distilled water to a depth of 2.5 cm. The mixture was then brought to boiling and then simmered (85–95°C) for 25 min. After sweet potatoes were fully cooked, they were drained and allowed to cool to room temperature. The sample was then blended in a food processor (2.5Qt Pro Commercial, Waring, McConnellsburg, PA) for 1 min. The homogenised potatoes were transferred to zip-top storage bags, frozen, and then freeze-dried (FreeZone Tray Dryer, Labconco, Kansas City, MO) before storage at −80°C for further use.

Red kidney beans were cooked as previously described (Rose et al., 2021) with some modifications. In particular, dry red kidney beans were soaked in distilled water at 1:5 (w/v) for 16 h at room temperature. The distilled water was then discarded, and the soaked kidney beans were transferred to a pot filled with fresh distilled water (1:10, w/v). The mixture was then brought to boiling and then simmered (85–95°C) for 1 h. After cooking, kidney beans were drained and blended in a food processor (Waring) for 1 min. The homogenised beans were transferred to zip-top storage bags, frozen, and then freeze-dried before storage at −80°C for further use.

The protein, starch, and dietary fibre in the cooked and freeze dried samples was measured using standard methods (AACC, 1999a, 1999b, 1999c). The kidney beans and sweet potatoes contained (mean ± standard deviation): 21.5 ± 0.1% and 3.6 ± 0.1% protein, 43.6 ± 1.0% and 60.0 ± 1.3% starch, and 21.6 ± 0.4% and 5.7 ± 0.2% dietary fibre, respectively.

### In vitro digestion and fermentation

Freeze-dried sweet potatoes and kidney beans were subjected to *in vitro* digestion as described (Bengtsson et al., 2009), with some modifications. Briefly, 3 g of freeze-dried sample was weighed into a 50 mL centrifuge tube. Then, 10 mL of simulated salivary fluid (50 mM NaCl, 10 mM KH_2_PO_4_, 2 mM CaCl_2_·2H_2_O, 40 mM NaHCO_3_ containing 1 mg/mL α-amylase [1000 U/mg, P-A6255, Sigma-Aldrich, St. Louis, MO]) were added, and the pH was adjusted to 6.7 with 1M NHCO_3_. The slurry was incubated for 15 min at 37°C in a water bath with reciprocal shaking at 100 rpm. Next, the pH was reduced to 2 with 1 M HCl before adding 5 mL of simulated gastric fluid (50 mM NaCl, 14 mM KCl, 3.5 mM KH_2_PO_4_, 10 mM CaCl_2_·2H_2_O, 3.6 mM MgCl_2_·6H_2_O, containing 21 g pepsin /L [914 U/g solids, P-7000; Sigma-Aldrich, St. Louis, MO]) and the mixture was incubated for 30 min at 37°C with shaking at 100 rpm. To simulate the intestinal phase, the pH was raised to 6.9 with 1M NHCO_3_, then 3 mL of pancreatin/bile solution (4.5 g/L pancreatin [P-7545; Sigma-Aldrich] and 28 g/L bile salts [Oxoid, Cheshire, England] in 100 mM NaHCO_3_) were added and the mixture was incubated for 2 h at 37°C with orbital shaking at 100 rpm. Following digestion, the slurry was transferred into dialysis tubing (MWCO 100–500 Da; Spectra Por 131060) and dialyzed for 72 h in distilled water at 4°C that was changed every 3 hours during the day (4 times/day). The retentate was then freeze-dried and stored at −80°C.

*In vitro* fermentation was performed as previously described (Yang and Rose, 2014) with some modifications. Briefly, inside the anaerobic chamber, 40 mg of freeze-dried beans or sweet potatoes obtained after *in vitro* digestion and dialysis were suspended in 4 mL of sterile anaerobic fermentation media in a Hungate tube. The fermentation medium contained (per L): 2 g peptone (Fisher Scientific, Waltham, MA), 2 g yeast extract (Fisher Scientific, Waltham, MA), 0.5 g bile salt (Oxoid, Cheshire, England), 2 g NaHCO_3_, 0.1 g NaCl, 0. 5g l-cysteine (Fisher Scientific, Waltham, MA), 2 mL Tween 80 (Fisher Scientific, Waltham, MA), 1 mL Vitamin K solution (10 μL/1 mL dissolved in ethanol; Alfa Aesar, Haverhill, MA), 4 mL of resazurin solution (1 mg/4 mL dissolved in water; Alfa Aesar, Haverhill, MA), 0.01 g MgSO_4_.7H_2_O, 0.01 g CaCl_2_·2H_2_O, 1 mL hemin solution (0.015 g hemin dissolved in 3 mL DMSO), and 0.04 g K_2_HPO_4_. Hungate tubes were then inoculated with 0.4 mL of faecal slurry in triplicate, and immediately sealed with a rubber stopper and aluminium seal. Each sample was inoculated with a faecal microbiome from one donor; no pooling of faecal samples was done. Then, tubes were incubated in a water bath at 37°C with orbital shaking at 60 rpm. After fermentation, samples were aliquoted in 1.5 mL centrifuge tubes and stored at −80°C. All fermentations were performed in triplicate with separate tubes for 0 h, 24 h, and 48 h measurements.

### Gas production

The gas volume produced during fermentation was measured after 24 h and 48 h by inserting a needle attached to a glass syringe through the septum of the fermentation tube and reading the gas volume from the graduations on the syringe. Gas volume was measured before opening the tube and aliquoting fermentation slurry for analysis of SCFA and microbiota composition.

### Short chain fatty acid analysis

The fermented samples were thawed on ice and centrifuged at 9600*g* for 10 min. The supernatants were then collected and used for SCFA analysis as previously described (Hartzell et al., 2013). In short, 0.4 mL of supernatant was vortex mixed with 0.1 mL of 7 mM 2-ethylbutyric acid in 2 M potassium hydroxide, 0.2 mL of 9 M sulphuric acid, and ~ 0.1 g of sodium chloride in a 2 mL screw cap microcentrifuge tube. Diethyl ether (0.5 mL) was then added, and the mixture was inverted several times followed by centrifugation at 13600*g* for 1 min. The top layer was collected and injected into a gas chromatograph (Clarus 580; PerkinElmer, Waltham, MA) equipped with a capillary column (Nukol; 30 m [l] × 0.25 mm [i.d.] × 0.25 μm [film thickness]; Supelco, Bellefonte, PA) and a flame ionisation detector. The quantification of SCFA was done by calculating response factors for each SCFA (acetate [A-6283, Sigma-Aldrich], propionate [49916, Honeywell, Charlotte, NC], and butyrate [108111000, Acros Organics, Geel, Belgium]) relative to 2-ethylbutyric acid (149411000, Acros Organics) using injection of pure standards.

### Microbiota composition

Bacterial DNA was extracted from bacterial pellets obtained from SCFA analysis using the BioSprint 96 workstation (Qiagen, Germantown, MD), Biosprint 96 One-For-All Vet kit, stool lysis buffer ASL (Qiagen, Germantown, MD), and bead beating. The amplicon sequencing of the V4 region of the bacterial 16S rRNA gene was completed using the Illumina MiSeq platform and the MiSeq reagent kit v2 (2 × 251 bp) (Kozich et al., 2013). Sequences were demultiplexed and barcodes were removed prior to sequence analysis with QIIME 2 (Bolyen et al., 2019). Sequence quality control, trimming, chimera removal, and denoising were performed with DADA2 (Callahan et al., 2016). Forward and reverse reads were truncated to 245 and 160 bp, respectively, to maintain sequence qualities above a pH red score of 30. Using DADA2, sequences were dereplicated into 100% ASVs for exact sequence matching. Taxonomy was assigned using the SILVA database (Quast et al., 2013). There were a total of 5112124 reads across 206 samples with a median sequencing depth of 25068.5 reads/sample. Samples were rarefied to a sequencing depth of 10166 reads/sample, which removed 3 samples due to low number of reads (2 faecal sample measurements from different microbiomes and one fermented sample from 48 h of fermentation of a Me_ND microbiome on potatoes; each of these samples still had two replicates for the analysis). Rarefying and diversity calculations (observed ASVs and Shannon index) were performed using the phyloseq package in R (version 4.1.3) (McMurdie and Holmes, 2013).

### Fermentation with M. eldenii isolates

Two *M. elsdenii* isolates were used to determine gas production during fermentation of sweet potatoes and acetate as substrates. *M. eldenii* 2FL 0620 M7 was provided as a gift from Synbiotic Health (Lincoln, NE, https://synbiotichealth.com/) and was isolated from a healthy human stool sample. The strain was isolated on de Man, Rogosa and Sharpe (MRS) agar (BD Difco, Franklin Lakes, NJ) following stepwise faecal fermentations as described (Kok et al., 2019). The classification of this isolate as *M. eldenii* was confirmed by Sanger sequencing, performed by a commercial provider (MCLAB, South San Francisco, CA). *M elsdenii* B159 was purchased from ATCC (17752) and was previously isolated from the rumen of cattle (Gutierrez et al., 1959; Rogosa, 1971).

To grow *M. elsdenii* isolates, modified Reinforced Clostridial Broth (RCB) containing (per L) 10 g peptone (Fisher Scientific, Waltham, MA), 10 g beef extract (Fisher Scientific, Waltham, MA), 10 g yeast extract (Fisher Scientific, Waltham, MA), 5 g dextrose, 5.0 g NaCl, 1 g soluble starch, 0.5 g l-cysteine HCl (Fisher Scientific, Waltham, MA), and 3 g sodium acetate was prepared, adjusted to pH 6.8 and autoclaved. When solidified medium was needed, 15 g/L agar (BD Bacto agar, P-214010, MD) was added to the growth broth, autoclaved, and poured onto agar plates.

To activate the freeze-dried *M. elsdenii* B159, the product ampoule was opened in an anaerobic chamber and the culture pellet rehydrated with 0.5 mL pre-reduced growth broth. Then, the rehydrated culture was streaked onto agar plates and incubated anaerobically for 24 h at 37°C. When visible growth was detected, a single colony was picked, streaked, and incubated a second time. Then, a single colony was picked and enriched anaerobically in 10 mL growth broth for 24 h at 37°C. Finally, frozen 20% glycerol stocks were prepared from this enriched *M. elsdenii* B159 culture. *M. eldenii* 2FL 0620 M7 was already provided as a frozen stock and was used directly.

Both *M. eldenii* 2FL 0620 M7 and B159 strains were separately grown anaerobically from frozen stock on RCB agar plates for 24 h at 37°C. Single colonies were inoculated into RCB broth and incubated anaerobically overnight at 37°C. Cell density of overnight cell cultures was measured by obtaining optical density at 600 nm. *M. eldenii* 2FL 0620 M7 and B159 isolates were cultured in RBC broth to a final density of 2.24 × 10^7^ CFU/mL and 1.9 × 10^8^ CFU/mL, respectively.

### Data analysis

All data were analysed using R (version 4.1.3) and RStudio (2022.02.3 Build 492) with various packages as described. To characterise faecal samples selected for this study, means of technical replicates were calculated after rarefication. Then, the Bray–Curtis distance matrix was subjected to hierarchical cluster analysis using the complete linkage method and plotted using the “ggtree” package (Yu et al., 2017). Differences in observed ASVs and Shannon index between Me_D and Me_ND microbiomes were calculated using a Wilcoxon test. Differences in taxonomic composition of the Me_D and Me_ND faecal microbiomes were calculated using the “DESeq2” package (Love et al., 2014). Dietary variables obtained from faecal donors were adjusted to a 1000 kcal intake basis and then subjected to principal components analysis with scaling. Results were plotted using the “factoextra” package (Kassambara and Fabian, 2020). Gas production and SCFA were analysed using a three-way nested ANOVA, where time (0 h, 24 h, and 48 h), substrate (control, beans, potatoes) and microbiome nested within *M. elsdenii* group (Me_D, Me_ND) were the factors. Tukey’s test was performed to determine significant differences between levels of the substrate X *M. elsdenii* group interaction at each time point and between identical samples across time. *M. elsdenii* 4415 relative abundance was analysed using pairwise Wilcoxon tests among all substrate X *M. elsdenii* group levels by time and for all times by substrate X *M. elsdenii* group levels. The *p*-values from the pairwise tests were adjusted for family-wise error rate using the Holm-Bonferroni procedure. For microbiota composition of the fermented samples, β-diversity analysis was performed using constrained analysis of principal coordinates (CAP) biplot based on the Bray–Curtis distance matrix. The CAP model was microbiome nested within *M. elsdenii* group + substrate + time. Then, PERMANOVA analysis was conducted to examine whether the composition of Me_D microbiomes was different from Me_ND microbiomes, using the “vegan” package with 99 permutations (Oksanen et al., 2022). The vector for gas production was added to the CAP biplot by correlating gas production with Eigenvalues from the CAP analysis and using the correlation coefficients for CAP1 and CAP2 as the *X* and *Y* coordinates for the vector in the biplot. Multivariate Association with Linear Models 2 (MaAsLin2) was used to identify ASVs that were associated with gas production during fermentation (Mallick et al., 2021). The MaAsLin2 model included only fermented samples (24 h and 48 h) and treated gas production as a fixed effect and microbiome, substrate, and time as random effects. Data from the *M. elsdenii* isolates experiments were analysed using a one-factor ANOVA by isolate followed by Tukey’s test to determine significant differences among substrates by isolate. ANOVA, correlations, and hierarchical clustering were performed using base R functions. Tukey HSD and Wilcoxon tests were calculated using the “rstatix” package (Kassambara, 2020). Results were plotted using the “ggplot2,” “cowplot,” and “ComplexHeatmap” packages in R (Gu et al., 2016; Wickham, 2016; Wilke, 2020).

## Results

### Characteristics of faecal samples

**Figure 1.**
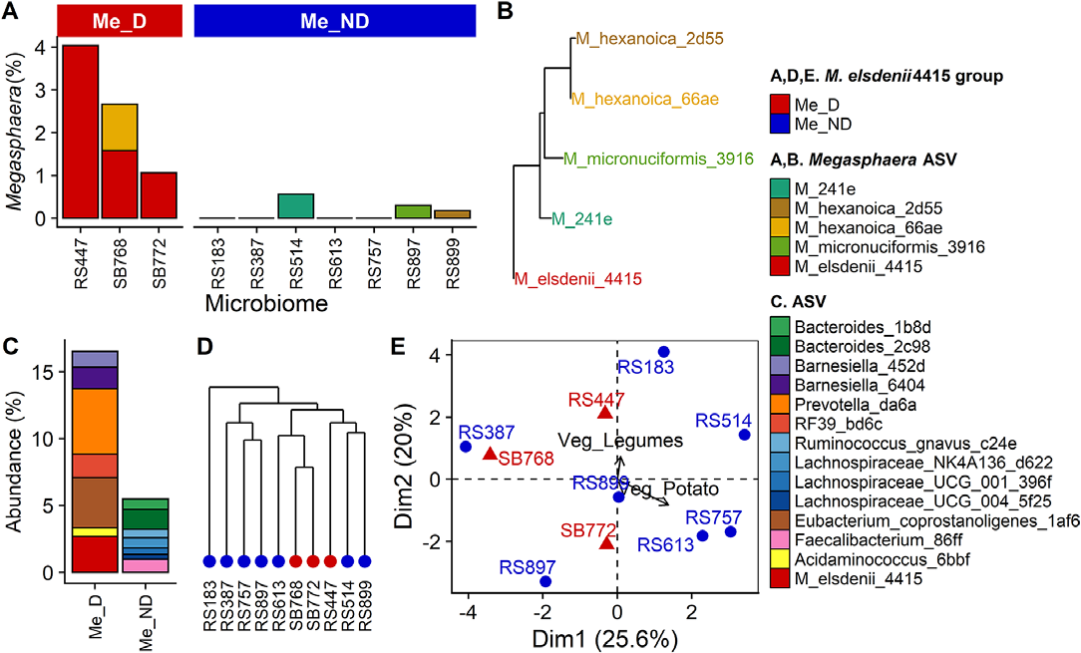
Characteristics of faecal microbiomes selected. *Megasphaera* ASV abundances in microbiomes with detectable *M. elsdenii* 4415 in faecal samples (Me_D) and with no detectable *M. elsdenii* 4415 in faecal samples (Me_ND) (A); phylogenetic tree of *Megasphaera* ASVs based on the V4 region of the 16S rRNA gene (B); differentially abundant ASVs (DESeq2 *p* adj < 0.05) (C); hierarchical clustering of Bray–Curtis distance matrix (D); principal components analysis biplot based on habitual intakes of faecal donors using 26 food categories (Supplementary Table S2) (E).

We identified three microbiomes from human stool (among 30) with detectable *M. elsdenii* 4415 (Me_D) (after rarefication to 10166 reads/sample; Supplementary Table S1). No other ASVs classified as *M. elsdenii.* The prevalence of *M. elsdenii* 4415 among the 30 microbiomes considered for this study (10%) was similar to previous reports on the occurrence of *M. elsdenii* in human stool (Sugihara et al., 1974). The abundance of *M. elsdenii* 4415 varied from 1.06% to 4.04% among the Me_D microbiomes (2.59 ± 0.86%; Figure 1A). Microbiomes RS447 and SB772 contained *M. elsdenii* 4415 as the sole ASV in the *Megasphaera* genus, while SB768 also contained *M. hexanoica* 66ae. Among the seven microbiomes selected to serve as the control group with no detectable *M. elsdenii* 4415 (Me_ND), four did not contain any *Megasphaera*, while the other three contained one unique *Megasphaera* ASV each. Among all *Megasphaera* ASVs identified in this data set, *M. elsdenii* 4415 ASV appeared to be phylogenetically distinct from the others (Figure 1B).

Although the abundance of *M. elsdenii* 4415 was significantly higher in the Me_D microbiomes compared with the Me_ND microbiomes, it was not the only ASV that was differentially abundant. There were six other ASVs from *Bacteroidota* and *Bacillota* that were significantly higher in the Me_D microbiomes, comprising a combined abundance of 16.5% (Figure 1C). Seven ASVs were significantly elevated in the Me_ND microbiomes compared with the Me_D microbiomes, also from *Bacteroidota* and *Bacillota*, but comprising only a combined abundance of 5.5%.

At the phylum level, the composition of the Me_D and Me_ND microbiomes were typical of human faecal samples, comprising a majority of taxa from *Bacteroidota* and *Bacillota* (Supplementary Figure S1). No differences at the phylum level were evident between Me_D and Me_ND microbiomes (DESeq2 *p*adj > 0.05).

Hierarchical clustering based on the Bray–Curtis distance matrix among faecal samples did not separate Me_D microbiomes from the Me_ND microbiomes (Figure 1D). The Me_D microbiomes fell into two different clusters containing other Me_ND microbiomes. Similarly, the α-diversity metrics, observed ASVs and Shannon index, did not differentiate Me_D from Me_ND microbiomes (observed ASVs, 124 ± 3 vs. 94 ± 10, Wilcoxon test *p* = 0.12; Shannon, 3.68 ± 0.12 vs. 3.44 ± 0.15, *p* = 0.27, respectively).

Because we were interested in gas production by the microbiomes during fermentation of two flatulogenic foods – sweet potatoes and kidney beans – we examined the habitual dietary intakes of the faecal donors using a food frequency questionnaire. A principal components biplot based on intakes of 26 food categories (servings per 1000 kcal) did not separate faecal donors by *M. elsdenii* group (Figure 1E). The categories that included intake of sweet potatoes (Veg_Potato) and kidney beans (Veg_Legumes) did not play a substantial role in separating subjects on the biplot (as indicated by the short vectors) and intake of these food groups were not significantly different between Me_D and Me_ND faecal donors (Veg_Legumes: 0.17 ± 0.16 cups/1000 kcal vs. 0.10 ± 0.04 cups/1000 kcal, Wilcoxon test *p* = 0.8; Veg_Potato: 0.06 ± 0.02 cups/1000 kcal vs. 0.13 ± 0.03 cups/1000kcal, *p* = 0.2).

### Gas production and M. elsdenii 4415 abundance during fermentation

**Figure 2.**
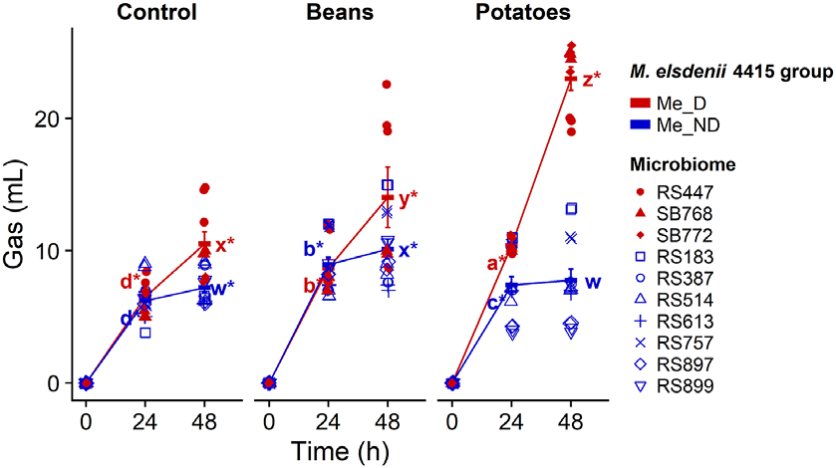
Microbiomes with detectable *M. elsdenii* 4415 in faecal samples (Me_D) produced more gas than microbiomes with no detectable *M. elsdenii* 4415 in faecal samples (Me_ND) during *in vitro* fermentation. Gas production during fermentation of control (no substrate), kidney beans (Beans), and sweet potatoes (Potatoes); error bars show standard error; different letters denote significant differences among samples at the same time point; * (asterisk) denotes significant differences from the corresponding sample at the previous time point (Tukey’s HSD *p* < 0.05).

The Me_D microbiomes produced significantly more gas than the Me_ND microbiomes after 24 h of fermentation of sweet potatoes, and, by 48 h of fermentation, the Me_D microbiomes produced more gas than the Me_ND microbiomes on both kidney beans and sweet potatoes (Figure 2). Me_D microbiomes that were treated with digested sweet potatoes as substrate produced significantly more gas than those treated with kidney beans at both 24 h and 48 h of fermentation, while the opposite was true for the Me_ND microbiomes.

**Figure 3.**
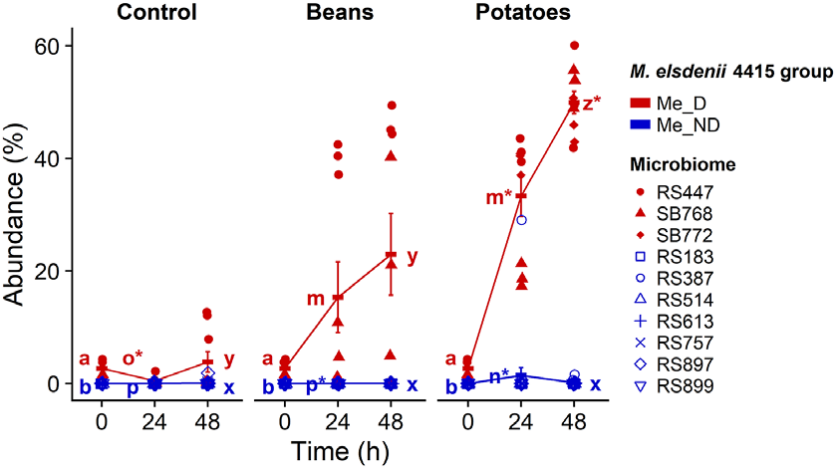
*M. elsdenii* relative abundance remained higher in *M. elsdenii* detectable (Me_D) microbiomes than *M. elsdenii* not detectable (Me_ND) microbiomes during *in vitro* fermentation. *M. elsdenii* relative abundance during 48 h of fermentation of control (no substrate), kidney beans (Beans), and sweet potatoes (Potatoes); error bars show standard error; different letters denote significant differences among substrates and *M. elsdenii* group at the same time point; * (asterisk) denotes significant differences from the corresponding sample at the previous time point (pairwise Wilcoxon test with Holm-Bonferroni-adjusted *p* < 0.05).

During fermentation, *M. elsdenii* 4415 relative abundances increased to up to 60% in some Me_D microbiomes (Figure 3). Although Me_ND microbiomes had no detected *M. elsdenii* 4415 in the stool samples (0 h of fermentation), *M. elsdenii* 4415 was detected after fermentation in some microbiomes in this group, but at a lower relative abundance (<2%, except one outlying replicate from RS387 at 24 h on sweet potatoes with 28% abundance) compared to Me_D microbiomes.

In addition to examining changes in *M. elsdenii* 4415 abundance during fermentation, we examined the temporal dynamics of all ASVs that were differentially abundant between Me_D and Me_ND groups in the faecal samples (i.e., ASVs in Figure 1C). Except for a significant increase in *Acidaminococcus* 6bbf at 24 h on kidney bean to 7.9% abundance, which did not increase further at 48 h, there were no other changes in ASV abundances over time during fermentation (Supplementary Figure S2). Furthermore, most ASVs stayed below 2% abundance during the course of the fermentation regardless of *M. elsdenii* 4415 group.

### Gut bacteria associated with gas production

**Figure 4.**
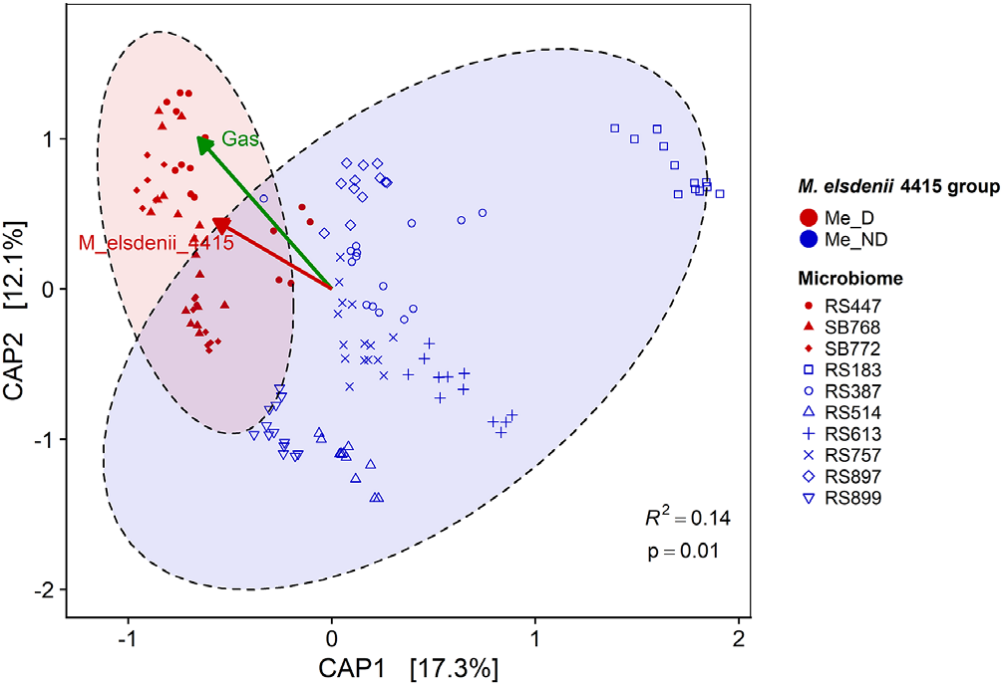
Microbiomes separated by *M. elsdenii* group and microbiomes with detectable *M. elsdenii* 4415 in faecal samples (Me_D) were associated with gas production. Constrained analysis of principal coordinates (CAP) biplot based on Bray–Curtis distance among fermented (24 h and 48 h) samples (*N* = 140). Eigenvector for *M. eldenii* 4415 as well as a vector for gas production calculated by correlating gas production with CAP scores for all samples are plotted; *R*^2^ and *p*-value shown are for the comparison between *M. eldenii* groups (Permutational Multivariate Analysis of Variance Using Distance Matrices); Me_ND, microbiomes with no detectable *M. elsdenii* 4415 in faecal samples.

A constrained analysis of principal coordinates biplot based on Bray–Curtis distances among samples showed that the microbiome composition of Me_D microbiomes was significantly different from Me_ND microbiomes during fermentation (Figure 4). The Eigenvector for *M. elsdenii* 4415 pointed towards the centre of the cluster of samples from the Me_D microbiomes, as expected. When gas production was overlaid on the plot, the direction of the vector showed that gas production was associated with the Me_D microbiomes and correlated with *M. elsdenii* 4415, but that there were also other taxa likely responsible for gas production during fermentation, especially in the Me_ND microbiomes.

**Figure 5.**
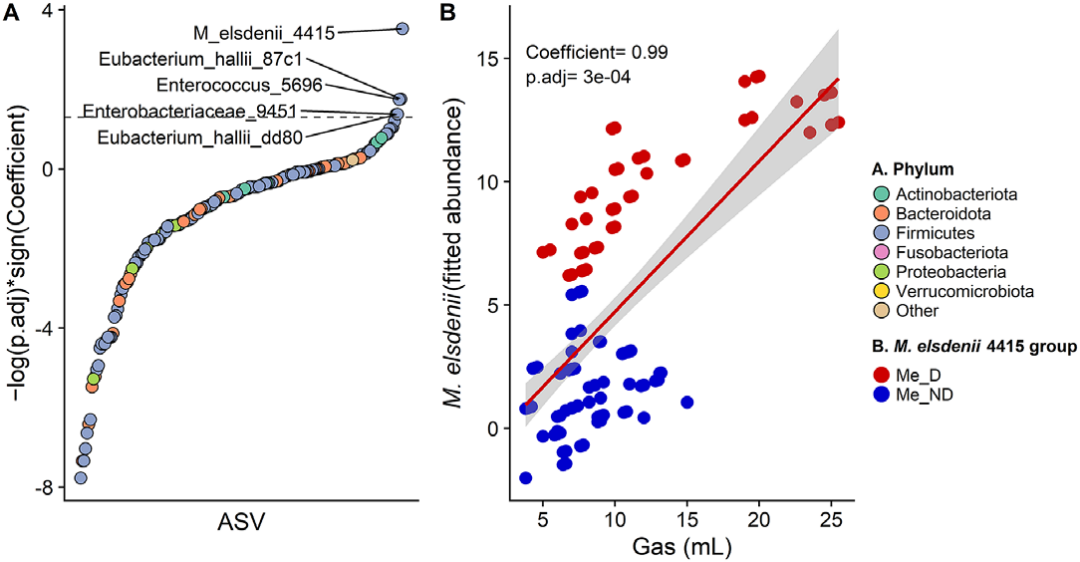
*M. elsdenii* 4415 abundance was the most strongly associated ASV with gas production during *in vitro* fermentation. Microbiome Multivariable Associations with Linear Models 2 (MaAsLin2) analysis of the association between ASV abundance and gas production (A); scatter plot of the relationship between gas production and *M. elsdenii* abundance (log transformed and adjusted for microbiome, fermentation time, and substrate by the MaAsLin2 model) (B); dashed line in panel A indicates the threshold for a significant positive relationship to gas production; Me_D/ND, microbiomes with detectable/no detectable *M. elsdenii* 4415 in faecal samples; *p.*adj, Benjamini-Hochberg-adjusted *p*-value.

MaAsLin2 was used to identify ASVs that were associated with gas production during fermentation of red kidney beans and sweet potatoes. Five ASVs were significantly positively associated with gas production (Figure 5A); however, *M. elsdenii* 4415 had the strongest association with gas production. A scatter plot of gas production versus fitted *M. elsdenii* 4415 abundances confirmed the strong correlation between *M. elsdenii* 4415 and gas production as well as the dramatic separation between Me_D and Me_ND microbiomes (Figure 5B).

When MaAsLin2 was run on only Me_D microbiome samples, *M. elsdenii* 4415 still maintained the strongest relationship to gas production (Supplementary Table S3); only one other ASV, *Olsenella* 94d1, was significant. In contrast, when only Me_ND microbiome samples were included in the analysis, 20 ASVs were significantly associated with gas production, and, not surprisingly, *M. elsdenii* 4415 was not included among these taxa. Among those ASVs that were significantly associated with gas production with all samples, *Enterococcus* 5696 and *Eubacterium hallii* dd80 (*Lachnospiraceae*) remained significant when only Me_ND samples were analysed. Two other ASVs from *Eubacterium* were also significant, as were five ASVs from *Bacteroides.*

### Short chain fatty acid production during fermentation

**Figure 6.**
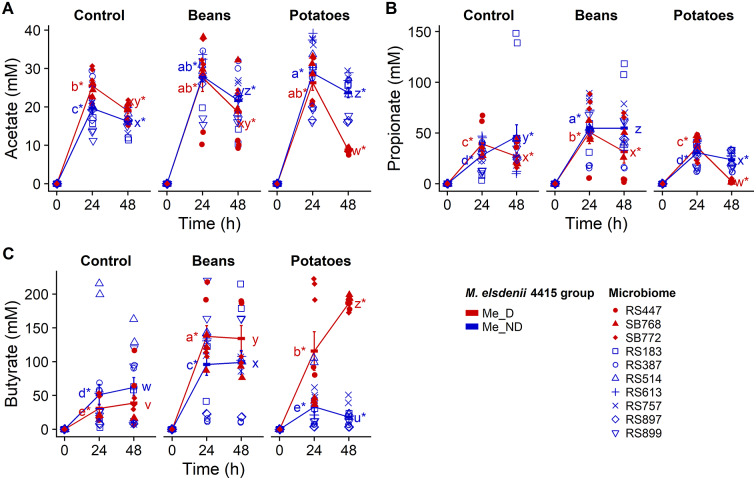
Microbiomes with detectable *M. elsdenii* 4415 in faecal samples (Me_D) were highly butyrogenic during *in vitro* fermentation. Acetate (A), propionate (B), and butyrate (C) production during 48 h of fermentation of control (no substrate), kidney beans (Beans), and sweet potatoes (Potatoes); Me_ND, microbiomes with no detectable *M. elsdenii* 4415 in faecal samples; error bars show standard error; different letters denote significant differences among substrates and *M. elsdenii* group at the same time point; * (asterisk) denotes significant differences from the corresponding sample at the previous time point (Tukey’s HSD *p* < 0.05).

After 48 h of fermentation, the Me_D microbiomes resulted in significantly more butyrate production compared to Me_ND microbiomes, particularly during fermentation of sweet potatoes but also during fermentation of kidney beans (Figure 6C). Interestingly, while acetate production was similar between the Me_D and Me_ND microbiomes at 24 h of fermentation, the Me_D microbiomes had a precipitous drop in acetate concentration between 24 h and 48 h of fermentation, while the Me_ND microbiomes showed a more tempered decrease in acetate concentration (Figure 6A). This decrease in acetate concentration corresponded to a steep increase in butyrate concentration – especially during fermentation of sweet potatoes – suggesting that there might be cross-feeding on acetate by microbiomes (and perhaps *M. elsdenii*) to produce butyrate. Propionate production was favoured in the Me_ND microbiomes compared to Me_D microbiomes during fermentation of both sweet potatoes and red kidney beans.

### Gas production from M. elsdenii isolates

**Figure 7.**
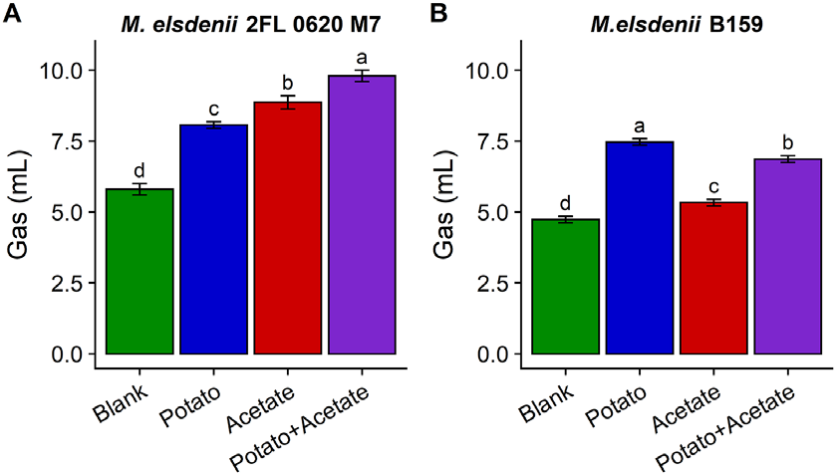
*M. elsdenii* 2FL 0620 M7 from human stool produced high quantities of gas from potatoes and acetate. Gas produced during fermentation of potatoes, acetate, or potatoes and acetate as carbon sources using *M. elsdenii* isolated from human stool (*M. elsdenii* 2FL 0620 M7) or the rumen of cattle (*M. elsdenii* B159); error bar shows standard deviation; different letters denote significant differences among substrates within isolate (Tukey’s HSD *p* < 0.05).

Two *M. elsdenii* isolates were separately grown with the same digested and dialyzed sweet potato substrate as used for the faecal fermentation experiments to determine if *M. elsdenii* would be able to metabolise sweet potatoes and produce gas in pure culture. Because the SCFA data suggested that there may be cross-feeding by gas-producers on acetate, acetate was also used as a substrate for fermentation as well as a combination of digested sweet potatoes and acetate. *M. elsdenii* 2FL 0620 M7, which was isolated from human stool, was able to produce relatively high volumes of gas directly from sweet potatoes, but produced even more gas during fermentation of acetate (Figure 7A). The most gas was produced when *M. elsdenii* 2FL 0620 M7 was supplied both potatoes and acetate. In contrast, *M. elsdenii* B159, which was isolated from the rumen of cattle, produced the highest volume of gas on sweet potatoes, but acetate was not well used by this isolate (Figure 7B). In fact, when acetate was included with potatoes, gas production was slightly, but significantly, diminished.

## Discussion

Flatulence is still a major deterrent for some people to consume dietary fibre-containing foods because of the association of these foods with increased gas production (Szczebyło et al., 2020). Consuming foods high in dietary fibre is not the only factor that induces gas symptoms; the gut microbiota is also responsible for differentiating gas production (Manichanh et al., 2014). In this study, we found that Me_D microbiomes produced more gas than Me_ND microbiomes during fermentation of sweet potatoes and red kidney beans. This is consistent with previous studies where *M. elsdenii* was significantly associated with gas production during the fermentation of pulses using human gut microbiota (Rose et al., 2021), grains using rumen microbiota (Meissner et al., 2014; Sedighi and Alipour, 2019), and purified fibres using horse microbiota (Douthit et al., 2019).

While only Me_D microbiomes had detectable *M. elsdenii* 4415 in faecal samples, which increased dramatically during fermentation, Me_ND microbiomes also showed a low abundance of *M. elsdenii* 4415 after fermentation even though it was not detected in the faecal samples. Thus, the high gas production contributed by *M. elsdenii* 4415 appears to be dependent on abundance, with sufficiently high relative abundance in stool samples (i.e., detectable) required to manifest the high gas production phenotype during fermentation. Given these findings, *M. elsdenii* 4415 is an important commensal member of the microbiome that is involved in elevated gas production during fermentation of gas-generating foods, but it is not a keystone species. Keystone species not only influence microbiome structure and function, but also do this irrespective of abundance across space and time (Banerjee et al., 2018). *M. elsdenii* 4415 fits the first part of the keystone species definition because it significantly affects gas production, but it fails to meet the second requirement because abundance also matters.

Although our primary focus was on the comparison of gas production between Me_D and Me_ND microbiomes during fermentation, there were some notable differences between red kidney beans and sweet potatoes in terms of gas production. Although kidney beans and sweet potatoes were selected based on their association to flatulence, most literature focuses on beans and other pulses as the most associated with gas production (Murphy et al., 1972; Fleming, 1981). Consistent with expectations, the Me_ND microbiomes did produce more gas on kidney beans than sweet potatoes; however, the opposite was true for the Me_D microbiomes. This emphasises the unusual behaviour of Me_D microbiomes in terms of gas production.

The differences in SCFA production – particularly butyrate and acetate – suggest that gas production during fermentation of sweet potatoes and kidney beans may be, at least in part, indirectly caused by metabolic cross-feeding between species that can ferment dietary fibres to acetate (or lactate) and gas-producing species that convert these acids to butyrate and gas (Kanauchi et al., 1999; Le Blay et al., 1999). *M. elsdenii* is primarily known as a lactate utiliser (Hino et al., 1994); however, it is also reported to be able to metabolise acetate (Forsberg, 1978; Counotte et al., 1981; Hino et al., 1991). Hino et al. (1991) showed that an *M. elsdenii* isolate from a ruminant animal (not specified) grew well on glucose, but growth was enhanced by addition of acetate. Interestingly, their study also showed that although growth was facilitated with the addition of acetate, hydrogen gas production was inhibited. These results are consistent with the inhibition of gas production seen during fermentation of sweet potatoes and acetate by *M. elsdenii* B159 from the rumen of cattle used in our study. In contrast, *M. elsdenii* 2FL 0620 M7 from human stool produced more gas on acetate than on sweet potatoes alone and the most gas on sweet potatoes and acetate together. Clearly, the relationship between acetate utilisation and gas production is different among *M. elsdenii* strains, and perhaps dependent on host species.

Evidently cross-feeding was very important to the high gas production in the Me_D microbiomes because the utilisation of acetate was very high and accompanied with higher butyrate production and gas. Therefore, possibly the Me_ND microbiomes did not have sufficient butyrate-producing bacterial species to use acetate for butyrate (and gas) production during fermentation. It has been reported that the rate and ratio of SCFA production depend upon the colonic microflora of an individual (Laurentin and Edwards, 2004). If the microbiome composition of an individual contains high counts of butyrate-producing bacterial species, in the presence of fermentable carbohydrates the conversion of acetate to butyrate will be quantitatively more significant (Morrison et al., 2006) and likely accompanied by high gas production (Yu et al., 2020).

The low propionate production in the Me_D microbiomes also provides support for the importance of acetate utilisation to produce gas and butyrate in these microbiomes. *M. elsdenii* is typically reported to be an important propionate producer in the rumen microbiome (Counotte et al., 1981; Hino et al., 1994). However, propionate is primarily produced during lactate utilisation; during fermentation of carbohydrates, *M. elsdenii* primarily produces butyrate (Shetty et al., 2013). Thus, during the fermentation of the kidney beans and sweet potatoes in this study, lactate utilisation was not a primary pathway used by *M. elsdenii* in the Me_D microbiomes; rather, *M. elsdenii* was using carbohydrates (and acetate). The substantial propionate produced by the Me_ND microbiomes could have been due to the elevated *Bacteroides*, which are important propionate-producing members of the microbiome (Blaak et al., 2020).

The unusual nature of the Me_D microbiomes was again emphasised in our analysis of individual taxa associated with gas production. In the Me_D microbiomes, gas production was driven primarily by *M. elsdenii* 4415, while under more “normal” conditions (i.e., in Me_ND microbiomes) gas production was generated by the metabolism of many taxa. For example, in the Me_ND microbiomes there were multiple ASVs from *Bacteroides* and *Eubacterium* (*Lachnospiraceae*) that were associated with gas production. *Bacteroides* is an important carbohydrate-using genus in the microbiota that typically uses carbon dioxide during metabolism of sugars, but several species, including those that degrade dietary fibres, produce hydrogen (Krieg et al., 2010). *Eubacterium* is an abundant genus in human faeces (Leitch et al., 2007). Several studies have reported that some bacterial species from this genus are associated with hydrolytic activities involved in the degradation of insoluble polysaccharides to produce hydrogen gas together with butyrate (Duncan et al., 2002; Leitch et al., 2007; Duncan and Flint, 2008; Flint et al., 2008). Cross-feeding on acetate (or lactate) may have also contributed to gas production from *Eubacterium.* In one study, when *E. hallii* was cocultured with *Bifidobacterium adolescentis* during fermentation of potato starch, *B. adolescentis* was shown to metabolise the substrate and produce lactate and acetate which was later used by *E. hallii* to produce butyrate (Belenguer et al., 2006).

Despite our compelling findings that a single taxon, *M. elsdenii* 4415, can have a dramatic influence on the volume of gas produced during fermentation of dietary substrates, our results reflect the production of gas during *in vitro* batch fermentation. *In vitro* fermentation experiments have been used extensively to study the direct effects of different gas-producing substrates on the gut microbiota without the confounding effects of other components in the diet or other covariates (Wang et al., 2019; Xie et al., 2019; Lu et al., 2020; Yu et al., 2020; Van den Abbeele et al., 2021). However, under *in vivo* conditions gases can be eliminated from the gastrointestinal tract via further metabolism by gas-consuming microorganisms, absorption into the bloodstream and exhalation via the breath, and through the anus as flatulence (Mego et al., 2015). Thus, flatulence and bloating are not only functions of the total gas volume produced, but also depend on the ability and rate of elimination of the gases by the microbiome and the gastrointestinal tract. Therefore, future studies should consider if our findings about *M. elsdenii* 4415 and gas production translate into symptoms of flatulence and bloating *in vivo.*

This study established that *M. elsdenii* is one commensal member of the microbiome that may be responsible for high gas production during the fermentation of flatulogenic foods. In contrast, gas production by microbiomes without detectable *M. elsdenii* was more complex and driven by a milieu of taxa. Gas production in microbiomes with detectable *M. elsdenii* was generated both directly through metabolism of dietary substrates, and indirectly through cross-feeding on acetate (and possibly lactate) produced by other commensal microorganisms. Elevated gas production from acetate was a characteristic of *M. elsdenii* isolated from human stool and not from ruminant animals.

## Supporting information

Mutuyemungu et al. supplementary material Mutuyemungu et al. supplementary material

## Data Availability

Raw sequence reads and metadata from faecal samples and *in vitro* fermentations, including gas production and short-chain fatty-acid data, are available in the NCBI Sequence Read Archive under accession no. PRJNA930037.

## Supplementary material

The supplementary material for this article can be found at https://doi.org/10.1017/gmb.2023.18.
