# Supplementary material for: Megasphaera elsdenii, a commensal member of the gut microbiota, is associated with elevated gas production during in vitro fermentation
Source: Gut Microbiome (Camb). 2023 Dec 21;5:e1. doi: 10.1017/gmb.2023.18 (PMC11406407; doi:10.1017/gmb.2023.18)
Supplement: Mutuyemungu et al. supplementary material [file S263228972300018Xsup001.docx]

**Table S1.** Megasphaera ASVs in 30 fecal samples considered in this study (relative abundance).

| Subject | Sex | Age |  | M. elsdenii 4415 group | M_elsdenii_4415 | M_hexanoica_2d55 | M_hexanoica_66ae | M_massiliensis_09db | M_micronuciformis_3916 | M_241e | M_6d7f | M_aae4 |
| --- | --- | --- | --- | --- | --- | --- | --- | --- | --- | --- | --- | --- |
| RS447 | M | 25 |  | Me_D | 4 | 0 | 0 | 0 | 0 | 0 | 0 | 0 |
| SB768 | M | 27 |  | Me_D | 1.6 | 0 | 1.1 | 0 | 0 | 0 | 0 | 0 |
| SB772 | F | 24 |  | Me_D | 1.1 | 0 | 0 | 0 | 0 | 0 | 0 | 0 |
| RS183 | F | 28 |  | Me_ND | 0 | 0 | 0 | 0 | 0 | 0 | 0 | 0 |
| RS387 | M | 23 |  | Me_ND | 0 | 0 | 0 | 0 | 0 | 0 | 0 | 0 |
| RS514 | F | 26 |  | Me_ND | 0 | 0 | 0 | 0 | 0 | 0.56 | 0 | 0 |
| RS613 | F | 25 |  | Me_ND | 0 | 0 | 0 | 0 | 0 | 0 | 0 | 0 |
| RS757 | F | 27 |  | Me_ND | 0 | 0 | 0 | 0 | 0 | 0 | 0 | 0 |
| RS897 | M | 21 |  | Me_ND | 0 | 0 | 0 | 0 | 0.3 | 0 | 0 | 0 |
| RS899 | M | 26 |  | Me_ND | 0 | 0.17 | 0 | 0 | 0 | 0 | 0 | 0 |
| SB765 | F | 30 |  | Not selected | 0 | 0 | 0 | 0 | 0 | 0 | 0 | 0 |
| SB766 | M | 28 |  | Not selected | 0 | 0 | 0 | 0 | 0 | 0 | 0 | 0 |
| SB767 | F | 37 |  | Not selected | 0 | 0 | 0 | 0 | 0 | 0 | 0 | 0 |
| SB769 | F | 23 |  | Not selected | 0 | 0 | 0 | 0 | 0 | 0 | 0 | 0 |
| SB771 | F | 60 |  | Not selected | 0 | 0 | 0 | 0 | 0 | 0 | 0 | 0 |
| SB775 | M | 41 |  | Not selected | 0 | 0 | 0 | 0 | 0 | 0 | 0 | 0 |
| SB776 | F | 27 |  | Not selected | 0 | 0.36 | 0 | 0 | 0 | 0 | 0 | 0 |
| SB777 | F | 27 |  | Not selected | 0 | 0 | 0 | 0 | 0 | 0 | 0 | 0 |
| SB778 | M | 28 |  | Not selected | 0 | 0 | 0 | 0 | 0 | 0 | 0 | 0 |
| SB779 | F | 58 |  | Not selected | 0 | 0 | 0 | 0 | 0 | 0 | 0 | 0 |
| RS134 | F | 29 |  | Not selected | 0 | 0 | 0 | 0 | 0 | 0 | 8.1 | 1.2 |
| RS238 | M | 40 |  | Not selected | 0 | 0 | 0 | 0 | 0 | 0 | 0 | 0 |
| RS259 | M | 28 |  | Not selected | 0 | 0 | 0 | 0 | 0 | 0 | 0 | 0 |
| RS279 | M | 41 |  | Not selected | 0 | 0 | 0 | 0 | 0 | 0 | 0 | 0 |
| RS316 | F | 30 |  | Not selected | 0 | 0 | 0 | 0 | 0 | 0 | 0 | 0 |
| RS403 | M | 25 |  | Not selected | 0 | 0 | 0 | 0 | 0 | 0 | 0 | 0 |
| RS441 | M | 26 |  | Not selected | 0 | 0 | 0 | 0 | 0 | 0 | 0 | 0 |
| RS582 | M | 29 |  | Not selected | 0 | 0 | 0 | 0.0049 | 0 | 0 | 13 | 0 |
| RS895 | M | 27 |  | Not selected | 0 | 0 | 0 | 0 | 0 | 0 | 0 | 0 |
| RS978 | M | 22 |  | Not selected | 0 | 0 | 0 | 0 | 0 | 0 | 0 | 0 |

**Table S2.** Food category intakes (mean ± standard error, servings/1000 kcal) of stool donors with detectable *M. elsdenii* (Me_D) and no detectable *M. elsdenii* (Me_ND) in fecal samples.^a^

| Food_group^b^ | Me_D | Me_ND | p.adj^c^ |
| --- | --- | --- | --- |
| Added_Fat | 13±3.5 | 15±1.7 | 1.00 |
| Added_Oils | 9±2 | 14±1.6 | 0.28 |
| Added_Sugar | 5.6±1.2 | 4.3±0.68 | 0.46 |
| Dairy_Cheese | 0.14±0.031 | 0.3±0.068 | 0.46 |
| Dairy_Milk | 1.7±0.5 | 0.28±0.085 | 0.14 |
| Dairy_Yogurt | 0.054±0.015 | 0.1±0.034 | 1.00 |
| Eggs | 0.41±0.32 | 0.42±0.12 | 0.83 |
| Fruit_CMB | 0.11±0.084 | 0.28±0.12 | 0.46 |
| Fruit_Juice | 0.045±0.028 | 0.1±0.032 | 0.62 |
| Fruit_Other | 0.34±0.23 | 0.38±0.055 | 0.83 |
| Grains_Ref | 2±0.38 | 2.2±0.29 | 0.94 |
| Grains_Whole | 0.81±0.58 | 0.61±0.17 | 0.75 |
| Prot_BPVLG | 0.17±0.053 | 1.1±0.23 | 0.14 |
| Prot_Cured | 0.053±0.012 | 0.45±0.068 | 0.14 |
| Prot_FishHi | 0.073±0.049 | 0.25±0.072 | 0.28 |
| Prot_FishLo | 0.097±0.067 | 0.55±0.21 | 0.28 |
| Prot_Nuts | 0.17±0.13 | 0.42±0.097 | 0.28 |
| Prot_Organ | 0.001±0.001 | 0.002±0.002 | 1.00 |
| Prot_Poultry | 0.35±0.17 | 0.74±0.09 | 0.28 |
| Prot_Soy | 0.23±0.15 | 0.16±0.1 | 0.75 |
| Veg_Green | 0.72±0.35 | 0.35±0.092 | 0.83 |
| Veg_Legumes | 0.17±0.16 | 0.097±0.044 | 0.94 |
| Veg_Other | 0.16±0.067 | 0.38±0.069 | 0.28 |
| Veg_Potato | 0.058±0.023 | 0.13±0.028 | 0.40 |
| Veg_RedOr | 0.031±0.003 | 0.13±0.033 | 0.22 |
| Veg_Tomato | 0.08±0.051 | 0.23±0.049 | 0.28 |

^a^ Intakes estimated from the Diet History Questionnaire III and converted to servings/1000 kcal.

^b^ Abbreviations: Added_Fat, Solid fat (g/100 kcal); Added_Oils, Oil (g/100 kcal); Added_Sugar, Added sugars (tsp/100 kcal); Dairy_Cheese, Cheese (cups/100 kcal); Dairy_Milk, Milk (cups/100 kcal); Dairy_Yogurt, Yogurt (cups/100 kcal); Eggs, Eggs protein foods (oz/100 kcal), Fruit_CMB, Citrus, melon, berry fruit (cups/100 kcal); Fruit_Juice, Juice fruit (cups/100 kcal); Fruit_Other, Other fruit (cups/100 kcal); Grains_Ref, Refined grain (oz/100 kcal); Grains_Whole, Whole grain (oz/100 kcal); Prot_BPVLG, Meat from beef, pork, veal, lamb, and game protein foods (oz/100 kcal); Prot_Cured, Cured meat protein foods (oz/100 kcal); Prot_FishHi, Seafood high in omega-3 protein foods (oz/100 kcal); Prot_FishLo, Seafood low in omega-3 protein foods (oz/100 kcal); Prot_Nuts, Nuts and seeds protein foods (oz/100 kcal); Prot_Organ Meat from organ meat protein foods (oz/100 kcal); Prot_Poultry, Poultry protein foods (oz/100 kcal); Prot_Soy, Soy products protein foods (oz/100 kcal); Veg_Green, Dark-green vegetable (cups/100 kcal); Veg_Legumes, Legumes vegetable (cups/100 kcal); Veg_Other, Other vegetable (cups/100 kcal); Veg_Potato, White potato starchy vegetable (cups/100 kcal); Veg_RedOr, Red/orange other vegetable (cups/100 kcal); Veg_Tomato, Red/orange tomato vegetable (cups/100 kcal).

^c^ Wilcoxon test with Benjamini-Hochberg adjusted p-value for comparison between *M. elsdenii* groups.

**Table S3.** ASVs that were significantly associated with gas production after correcting for microbiome, substrate, and time (MaAsLin2) using only samples with detectable *M. elsdenii* in fecal samples (Me_D) and no detectable *M. elsdenii* (Me_ND).

| data.set | ASV | coef | stderr | p | p.adj | N | N.not.zero |
| --- | --- | --- | --- | --- | --- | --- | --- |
| Me_D only | M_elsdenii_4415 | 1 | 0.28 | 0.00086 | 0.011 | 49 | 49 |
| Me_D only | Olsenella_94d1 | 0.9 | 0.26 | 0.0014 | 0.016 | 49 | 7 |
| Me_ND only | Eubacterium_hallii_aac0 | 1.1 | 0.28 | 0.00031 | 0.005 | 91 | 21 |
| Me_ND only | Phascolarctobacterium_1569 | 1 | 0.23 | 0.000025 | 0.0014 | 91 | 76 |
| Me_ND only | Veillonella_923f | 1 | 0.27 | 0.00032 | 0.005 | 91 | 52 |
| Me_ND only | Roseburia_ec67 | 0.99 | 0.22 | 0.000017 | 0.0013 | 91 | 17 |
| Me_ND only | Bacteroides_bf61 | 0.82 | 0.22 | 0.00041 | 0.0053 | 91 | 35 |
| Me_ND only | Eggerthella_629f | 0.81 | 0.27 | 0.0032 | 0.024 | 91 | 79 |
| Me_ND only | UCG_002_68d7 | 0.64 | 0.19 | 0.0015 | 0.015 | 91 | 31 |
| Me_ND only | Eubacterium_hallii_dd80 | 0.58 | 0.2 | 0.0081 | 0.044 | 91 | 21 |
| Me_ND only | Enterococcus_5696 | 0.56 | 0.14 | 0.00023 | 0.0045 | 91 | 28 |
| Me_ND only | UBA1819_f479 | 0.54 | 0.15 | 0.00036 | 0.0052 | 91 | 24 |
| Me_ND only | Lachnoclostridium_360b | 0.52 | 0.17 | 0.0022 | 0.02 | 91 | 16 |
| Me_ND only | Bacteroides_b151 | 0.46 | 0.15 | 0.0024 | 0.022 | 91 | 60 |
| Me_ND only | Bacteroides_plebeius_47c3 | 0.45 | 0.1 | 0.000052 | 0.002 | 91 | 16 |
| Me_ND only | Bacteroides_intestinalis_9d88 | 0.43 | 0.16 | 0.0092 | 0.046 | 91 | 16 |
| Me_ND only | Candidatus_Soleaferrea_4ac4 | 0.43 | 0.13 | 0.0016 | 0.016 | 91 | 13 |
| Me_ND only | Bacteroides_salyersiae_b7e5 | 0.42 | 0.14 | 0.0042 | 0.028 | 91 | 10 |
| Me_ND only | Raoultibacter_timonensis_4a42 | 0.41 | 0.14 | 0.0047 | 0.031 | 91 | 13 |
| Me_ND only | Ruminococcaceae_bacterium_f94b | 0.39 | 0.15 | 0.0087 | 0.046 | 91 | 14 |
| Me_ND only | Eubacterium_limosum_202f | 0.33 | 0.11 | 0.0041 | 0.028 | 91 | 12 |
| Me_ND only | Blautia_sp._02b4 | 0.29 | 0.094 | 0.0027 | 0.023 | 91 | 10 |

**
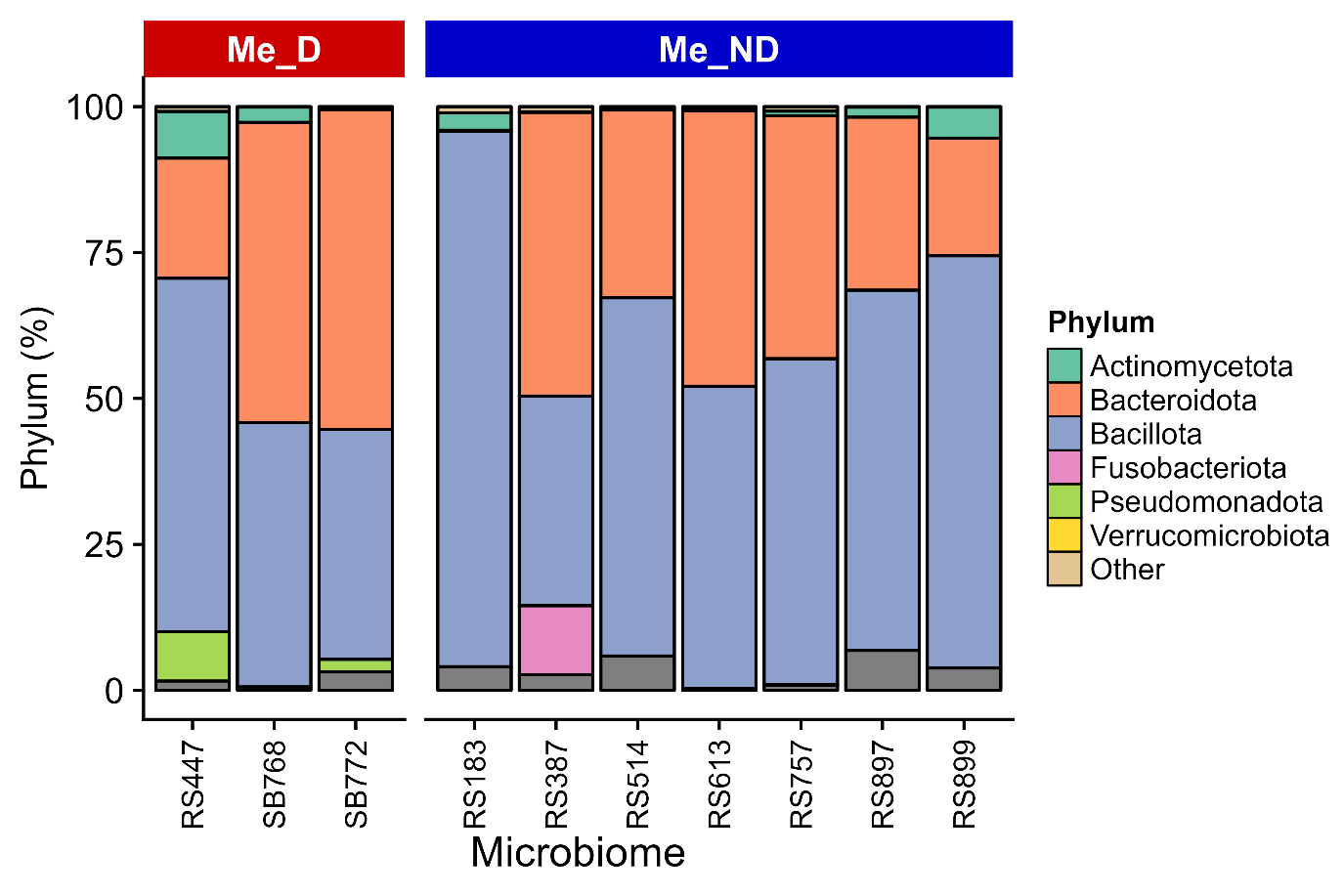
FIG S1** Phylum level abundances of fecal samples.


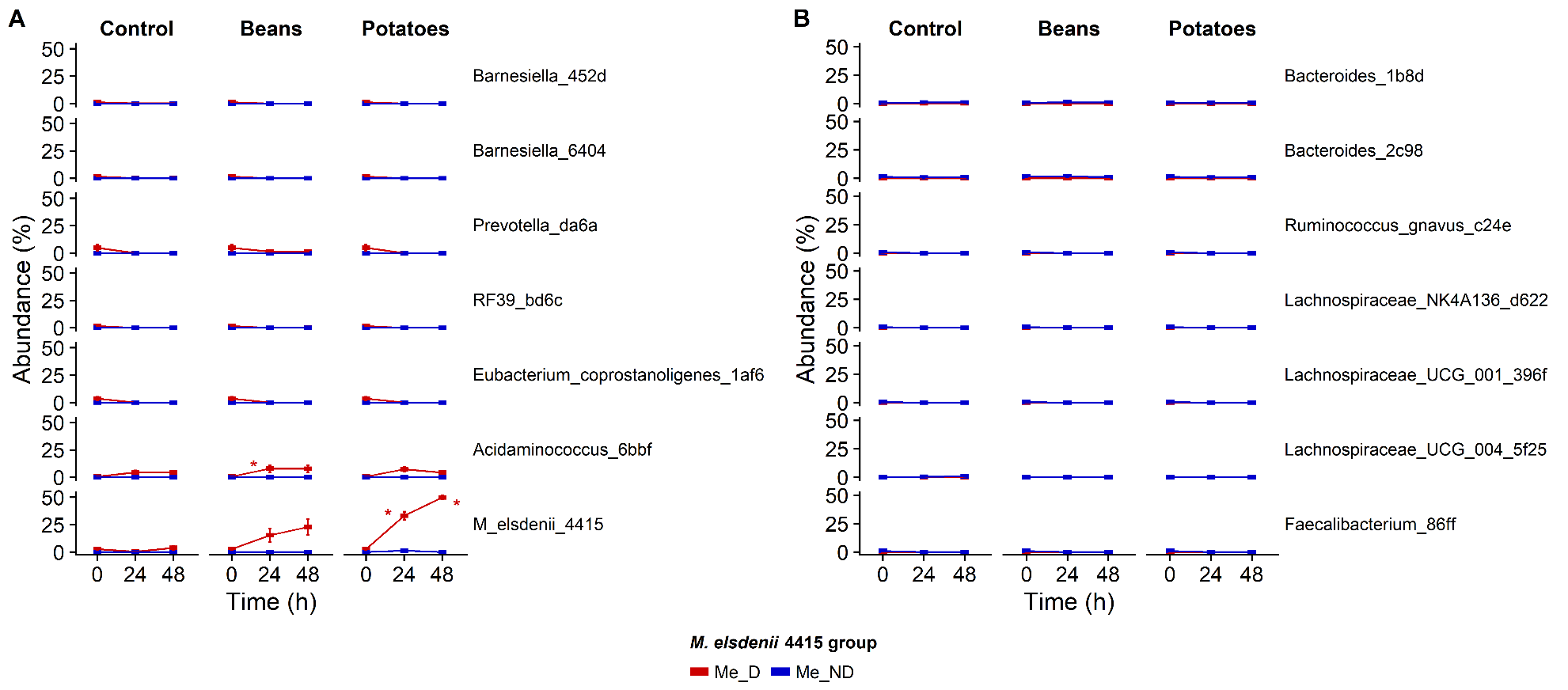
**FIG S2** Abundances of differentially abundant ASVs during fermentation. ASVs significantly higher in fecal samples of Me_D microbiomes (A); ASVs significantly higher in fecal samples of Me_ND microbiomes; * (asterisk) denotes significant differences from the corresponding sample at the previous time point (pairwise Wilcoxon test with Holm-Bonferroni-adjusted p<0.05)..
